# Using the Isolated Rat Placenta to Assess Fetoplacental Hemodynamics

**DOI:** 10.3389/ftox.2022.814071

**Published:** 2022-02-08

**Authors:** K. L. Garner, E. C. Bowdridge, E. DeVallance, J. A. Griffith, E. E. Kelley, T. R. Nurkiewicz

**Affiliations:** ^1^ Department of Physiology and Pharmacology, West Virginia University School of Medicine, Morgantown, WV, United States; ^2^ Center for Inhalation Toxicology (iTOX), West Virginia University School of Medicine, Morgantown, WV, United States

**Keywords:** placenta, validation, technique, protocol, reproduction, fetus, hemodynamics

## Abstract

Placental health is critical to fetal growth and maternal health during gestation. However, investigating placental flow in an *ex-vivo* isolated system where inflow is independently controlled has yet to be developed in the rat. Here, we describe a novel technique, isolated perfused placenta technique that allows for analysis of placental pressure outflow pressure, placental flow in rats at gestational day 20. Using this method, we successfully perfused placentas from dams and were able to observe increases in outflow pressure and flow as the inflow pressure to the placenta was increased in a step wise fashion. This method will help to advance the functional analysis of placental flow and therefore placental resistance and efficiency.

## Introduction

The rat has been long established as an ideal animal model to study both pregnancy and the fetoplacental interface due to its similarities with the human. Despite the fact that some differences exist between the two species, the function of the discoid placenta and the lineage of the cells comprising the maternal-fetal interface are overwhelmingly comparable (Soares et al., 2012). Additionally, the rat and human both have a hemochorial placenta in which only two fetal cell layers are crossed in the exchange of molecules between the dam and fetus (Soares et al., 2012). However, it should be noted that the rat has one layer of cytotrophoblast compared to two layers of syncytiotrophblast cells, whereas the human placenta has one only layer of each trophoblast cells type separating the maternal and fetal blood systems (Furukawa et al., 2019). Give that the similarities between the species, this model provides scientists with the opportunity to study physiological processes and challenges to homeostasis that would otherwise be ethically impossible in humans.

Fetal and placental weights at or near birth are positively corelated and are often used as an indicator of the ability of the placenta to provide the appropriate amount of nutrients to the fetus (Fowden et al., 2009). Placental efficiency (gram of pup per gram of placenta; Wilson and Ford, 2001), is often used as a metric of pregnancy health. Placental efficiency ranges from 5 g/g in humans to 20 g/g in horses (Fowden et al., 2009). Decreased placental efficiency is usually an indicator of poor placental vascularization that has occurred during gestation and/or insufficient maternal blood flow to the placenta. Conversely, deprivation of caloric or protein intake reduces oxidation substrates and may contribute to increased placental efficiency. Ultimately, a decrease in maternal blood flow or improper placental vascularization, lead to decreased placental transport of nutrients and oxygen exchange through flow-limited passive diffuse processes (Fowden et al., 2006). This may potentially contribute to smaller fetuses at term.

Environmental factors may also lead to decreased efficiency within the placenta, one of which being toxicant exposure during critical windows of gestational development. Considering the growing number of toxicants humans are exposed to throughout our lifetime, safety assessment of these known toxicants, the doses and discovery of new toxicants, is critical to improving fetal and maternal health. Therefore, a need exists to be able to discreetly study placental physiology under tightly controlled conditions.

We have developed a rat model of gestational toxicant exposure, through which placental flow can be assessed. This model is unique in regard to assessment of hemodynamics within the placenta proper and does so without the involvement of any maternal vascular inputs. Other groups have successfully used an *ex vivo* placental perfusion model to study the transfer of chemical compounds from dam to fetus in rats (Fournier et al., 2020) and in humans (Conings et al., 2017). Placental flow and pressure are assessed by manipulating input pressure over the physiological range *via* the umbilical artery and measuring the output flow pressure delivered to the umbilical vein. Dissipation of pressure and flow rate across this circuit is a function of vascular resistance, which has active (umbilical artery and vein tone) and passive elements (anatomical structure within the placenta, vessel bifurcation). Both these elements can be assessed with this model. Using a modified pressure myography chamber, inflow pressure to the placental unit is independently controlled, and outflow pressure and placental flow are recorded. Further, this model allows for assessment of multiple placentas from the same dam and characterization of placental hemodynamics as determined by sex of the fetus, placement within the uterine horn, and many other factors. We present data herein that demonstrate our ability to modify this chamber, and reliability and successfully measure placental flow in rats.

## Materials and Equipment

**Equipment:** Isolated vessel chamber (CH-1; Living Systems; St. Albans, VT), pressure servo pump (PS-200-P; Living Systems; St. Albans, VT), pressure monitor (PS-200-S; Living Systems; St. Albans, VT), peristaltic pump (Masterflex L/S 77200-60; Living Systems Instrumentation, St. Albans, VT), size matched glass pipettes (∼300 μm; Living Systems; St. Albans, VT), dissecting dish, forceps, scissors, silk braided suture (9–0; Unify #S-S918R13), LabChart software (AD Instruments, Colorado Springs, CO). Livings Systems chambers were selected as this is a complete integrated system that can be easily adapted to the isolated placenta. CH-1 chambers were selected because the entire chamber can be enclosed and pressurized, and temperature controlled. LabChart Software was chosen because it has the ability to record the digital data output from the Living Systems equipment (pressure, flow, vessel diameter).

**Solutions:** Physiological salt solution (PSS, in mmol/L: 129.8 NaCl, 5.4 KCl, 0.5 NaH_2_PO_4_, 0.83 MgSO_4_, 19.0 NaHCO_3_, 1.8 CaCl_2_, 5.5 glucose), Calcium free-PSS (Ca^2+^-free).

**Drugs:** S-nitroso-N-acetyl-d,l-penicillamine (SNAP).

## Methods

**FIGURE 1:**
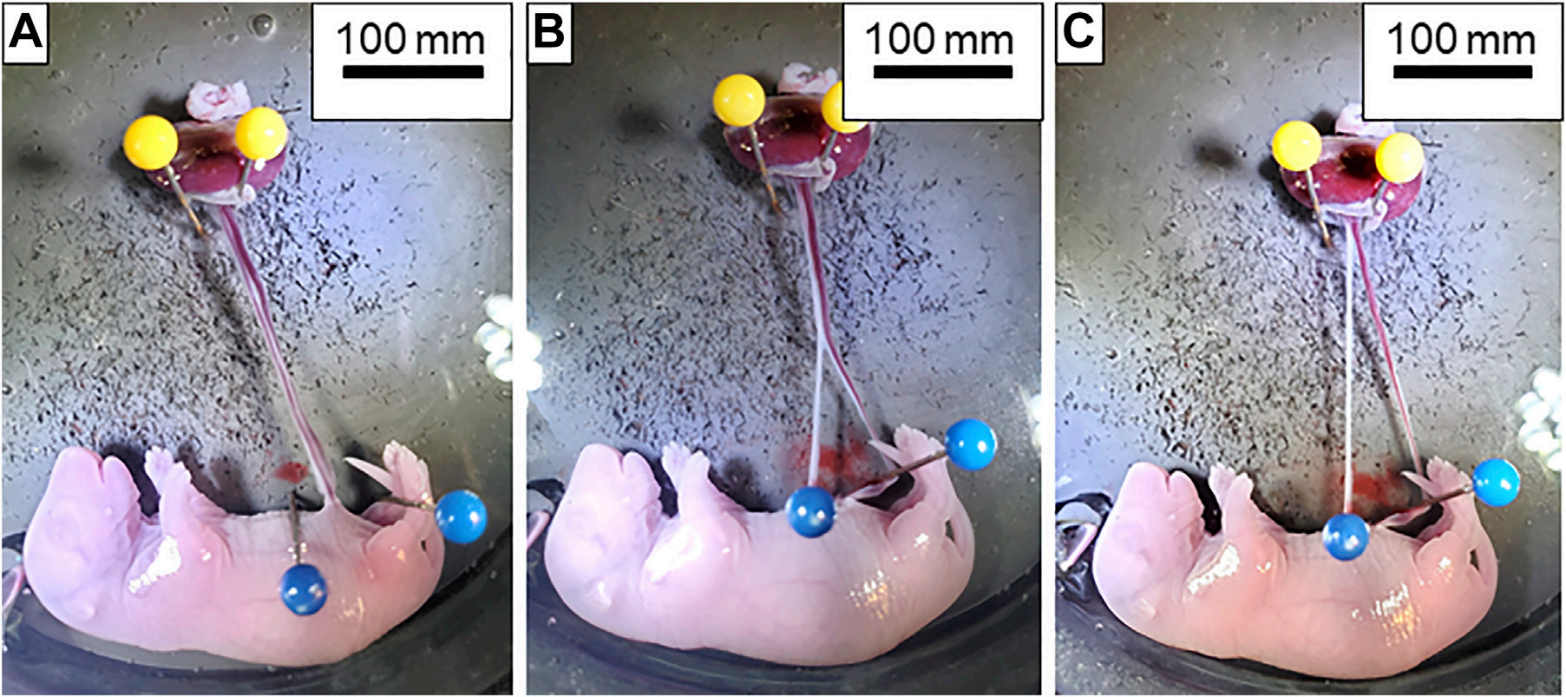
Dissection of the umbilical artery and vein. **(A)** Isolated fetoplacental unit with the umbilical artery and vein completely intact. **(B)** Dissection of the umbilical artery (white vessel located on the left) and vein (red vessel on the right) beginning at the insertion into the fetus. **(C)** Umbilical artery and vein completely separated from fetus to the placenta and ready for transfer to the chamber.

**FIGURE 2:**
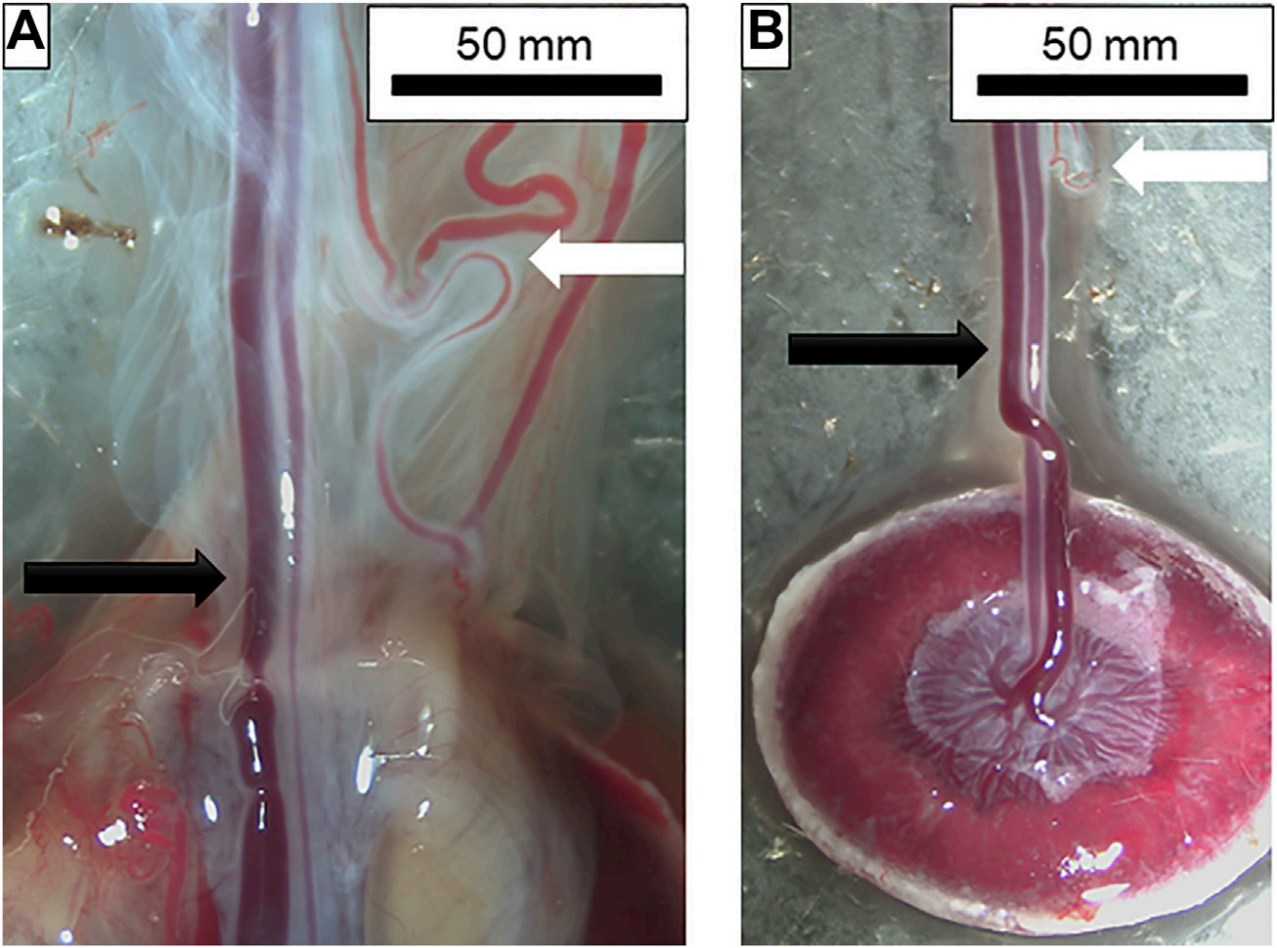
Dissection of placental and chorionic vessels. **(A)** From left to right: umbilical vein; umbilical artery; vitelline artery and other chorionic vessels. Amnionic membranes can also be seen surrounding the vessels prior to removal. Black arrow indicates the umbilical artery and vein pair while the white arrow shows the vitelline artery and chorionic vessels. **(B)** Placental unit with membranes removed and umbilical artery and vein isolated. Note the chorionic vessels with the white arrow at the top of the figure indicating how closely these two are prior to dissection. Vessel insertion and branching into the placenta can also be seen.

**FIGURE 3:**
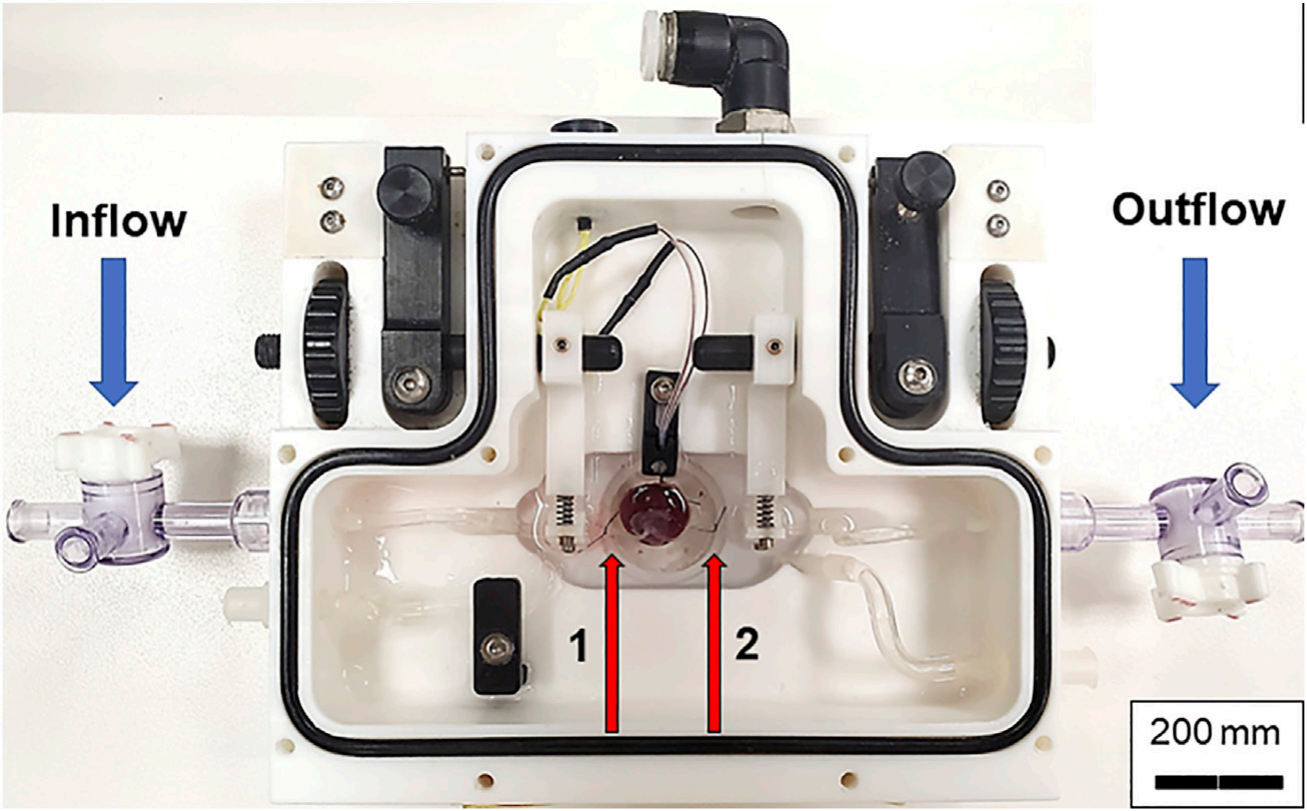
Chamber set up for isolated placental perfusion. The placental unit with separated umbilical artery and vein is placed into the Living Systems chamber with PSS. The umbilical artery is canulated onto the inflow glass pipette while the umbilical vein is canulated onto the outflow pipette and tied with braided silk suture. Red arrows indicate the inflow (left, 1) and outflow (right, 2) pipettes.

**FIGURE 4:**
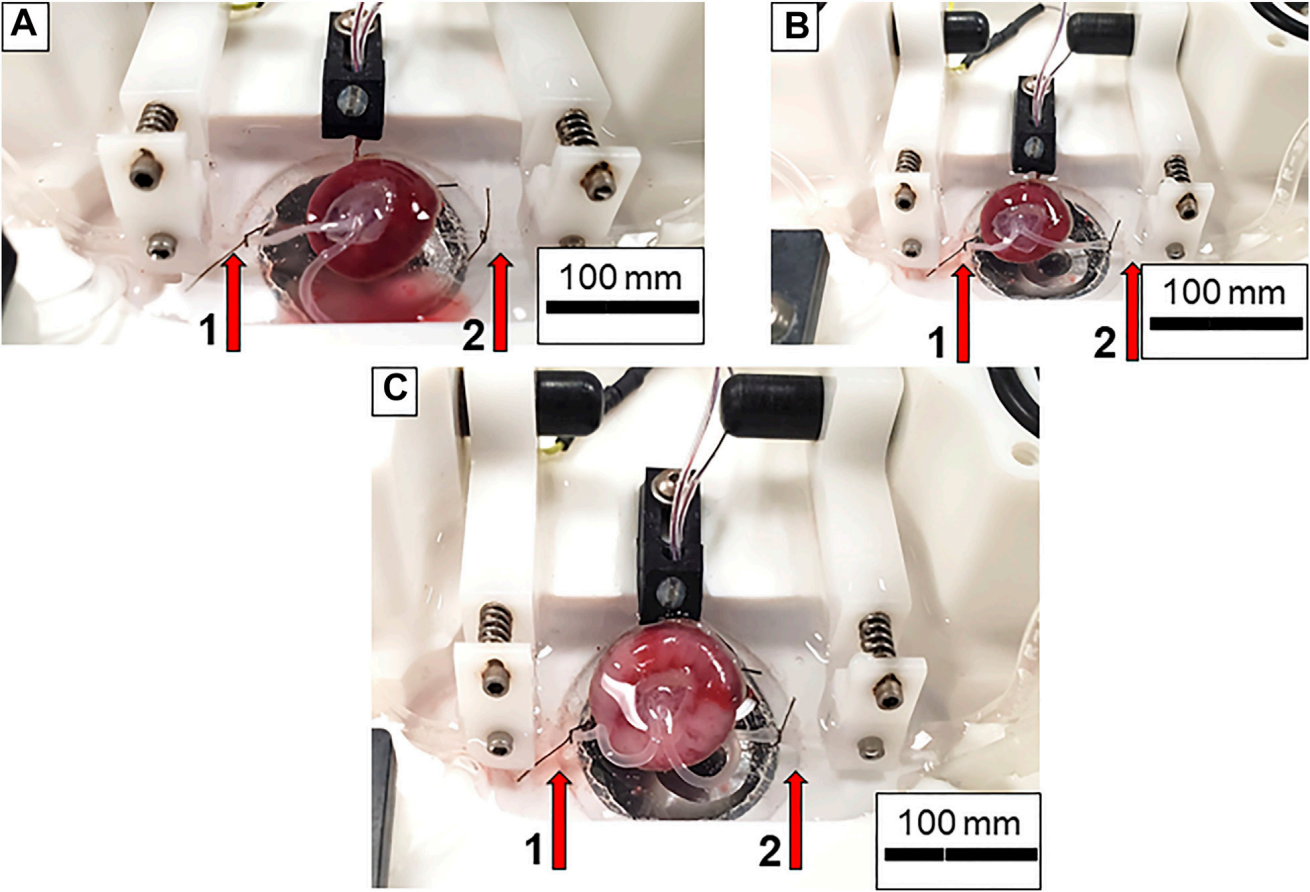
Preparation of the placental unit prior to starting an experiment. **(A)** The umbilical artery is canulated onto the inflow pressure canula while the umbilical vein is left united in order to clear the blood from the placenta. Note the pooling blood in the bottom of the chamber as PSS is flowing through the placenta. **(B)** Both umbilical artery and vein are placed onto and tied to the inflow and outflow cannulas, respectively. No pressure is being applied to the system. **(C)** The placenta is fully pressurized. Note the increase in size and volume of the vessels within the chamber. Again, red arrows indicate inflow (left, 1) and outflow (right, 2) pipettes.

Pregnant Sprague Dawley rats (Hilltop, Scottdale, PA) at gestational day (GD) 20 are anesthetized under general anesthesia (isoflurane gas, 5% induction, and 2% maintenance) and placed on a heating pad to maintain at 37°C rectal temperature. The uterus is harvested and placed in Ca^2+^-free PSS (4°C) in a chilled circulation dish (Preparatory Tissue Bath, #158401; Radonti; Covina, CA) and maintained at this temperature with a circulating chiller (IsoTemp 3016D; Fisher Scientific). The left and right uterine horns are pinned out. A tissue section containing the fetus, placenta, uterine wall and supplying vasculature are surgically excised and transferred to a clean dissection dish. The uterine wall is cut longitudinally, and the umbilical cord is cut as close to the fetus as possible immediately prior to mounting the placenta in the chamber. All uterine vascular conduits are ligated with 9–0 silk suture to contain perfusate flow exclusively to the fetal side of the placenta and maintain intervillous volume/pressure (Figures 1A–C). Ligating all the maternal vascular inputs is one unique aspect of this placental flow model and assures that any changes in flow that are observed are due to hemodynamic alterations within the placenta. The vitelline artery is removed, and the umbilical cord is opened to reveal the paired umbilical artery and vein. The umbilical artery and vein are gently dissected away from one another from the umbilical stalk of the fetus (Figure 1A) up to the insertion point of the vessels into the placenta (Figure 1C). A magnified view of the distinction between the umbilical artery and vein compared to the vitelline artery and chorionic vessels can be seen in Figure 2. Connective tissue is gently dissected/cleaned, and the artery is mounted on a size-matched pipette (∼300 µm). The umbilical artery is placed onto the inflow pipette and securely tied with 9–0 silk braided suture (Figure 3). The umbilical vein is left unmounted so that residual blood within the placenta can be gently flushed under low pressure prior to starting the experiment (Figure 4A). This step is critical to obtain accurate flow readings throughout the experiment. Once the blood has been removed from the placenta the umbilical vein is securely tied to the outflow pipette (Figure 4B). The tissue preparation is now ready for study using the Living Systems chambers (St. Albans, VT) and measurement of inflow and outflow pressure as well as placental flow using LabChart software (Colorado Springs, CO). The completed preparation is presented in Figure 4C. The chamber is maintained at 37°C, and the perfusate and superfusate are continuously bubbled with 21% O2–5% CO2. To begin the experiment, inflow pressure within the umbilical artery is increased in a stepwise fashion by 5 mm Hg, starting at 0 mm Hg, until maximum pressure is reached (∼40–60 mm Hg). At each incremental increase in pressure a steady state outflow pressure is reached (approximately 2 min) prior to the subsequent increase in pressure. We typically experience resistance across the placenta where outflow pressure is approximately half of inflow pressure under normal superfusate conditions in control placentas. Any treatment administered to the dam prior to tissue harvest, or pharmacological treatment within the bath can affect this resistance. Once the initial pressure with PSS has been established as the baseline the bath is washed and treatment of the placenta with pharmacological agents of interest can be added and the incremental increase in inflow pressure repeated. The successfully prepared placental unit once dissected is viable for up to 4 h when kept on ice from the time of tissue removal from the dam. Viability is assessed as the ability to cannulate the umbilical artery and vein without tissue degradation as well as pressurize the placenta with resultant outflow measurements. Because maternal/fetal placental integrity begins to drop at the time of tissue harvest it is imperative to verify pressurization is being held in the preparation throughout the duration of the experiment. Observing an inability to maintain this indicates the experiment should be ended and the placenta is no longer viable. This has been repeatedly measured in our laboratory over the last 4 years when we began using this fetoplacental preparation in a variety of treatment groups at GD 20, and 4 h has been shown to be the maximum viability time to start an experiment from the time of euthanasia of the dam and removal of tissue. From the time of tissue removal to the end of an experiment, an experienced technician would be able to complete this procedure in 1.5–2 h. Therefore, the examination of multiple placentas from the same dam is possible once the technique is mastered allowing for more biological repetition and repeatability. Importantly, endothelium-dependent, endothelium-independent relaxation, and vascular smooth muscle contractility as well as mechanotransduction (flow/shear stress and transmural pressure/myogenic responsiveness) are all able to be assessed within this isolated placenta system. Additionally, the influence of local metabolic factors may be assessed by altering (O_2_), (CO_2_) and/or temperature.

## Results

**FIGURE 5:**
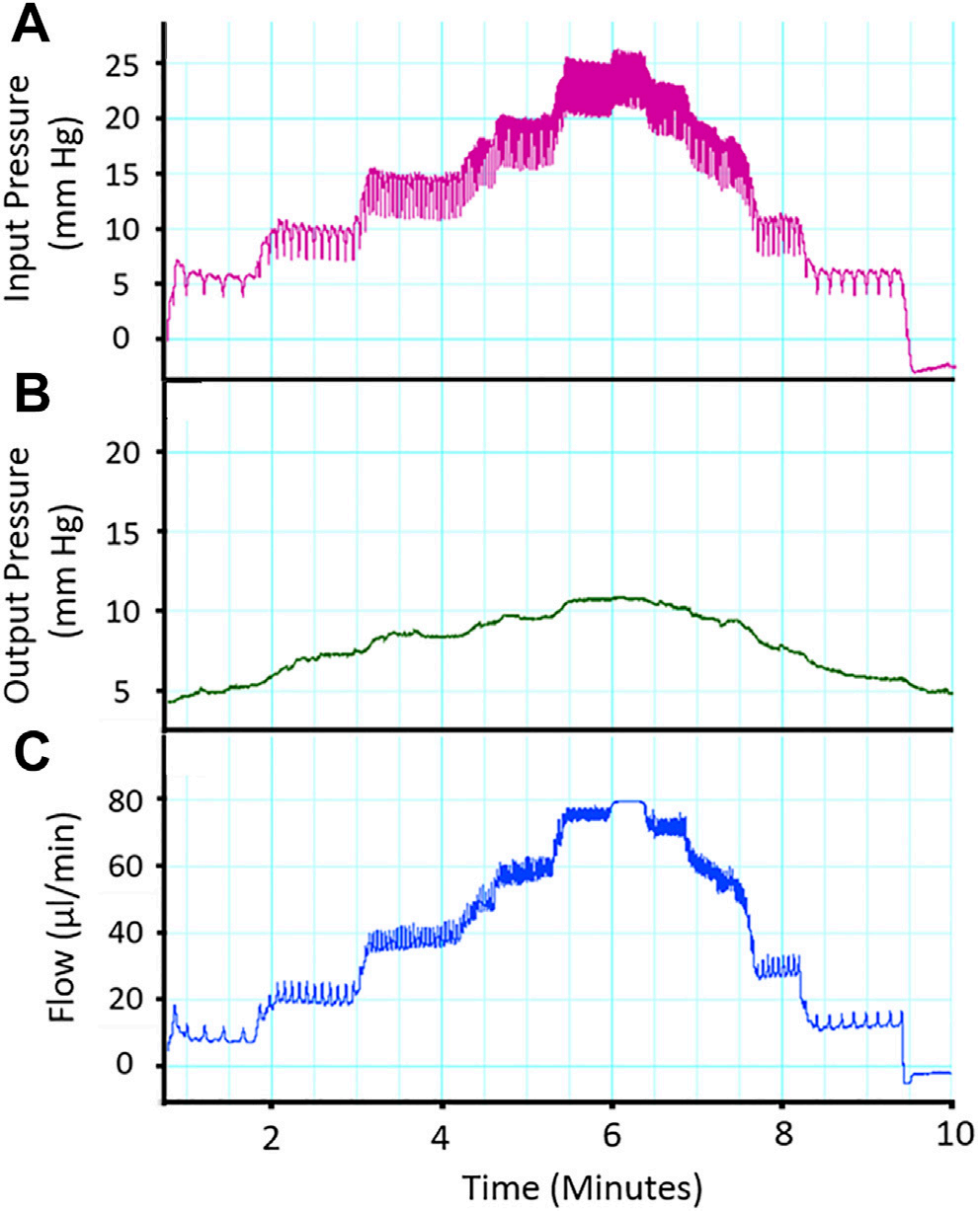
Representative trace of placental flow. **(A)** Input (umbilical artery) perfusion pressure is increased in a stepwise fashion to measure placental resistance. **(B)** Output pressure is measured and a drop in pressure across the placenta is expected and indicative of placental health. **(C)** Alterations in placenta flow (μL/min) can be seen concomitantly with the change in inflow and outflow pressure, indicating placental resistance.

**FIGURE 6:**
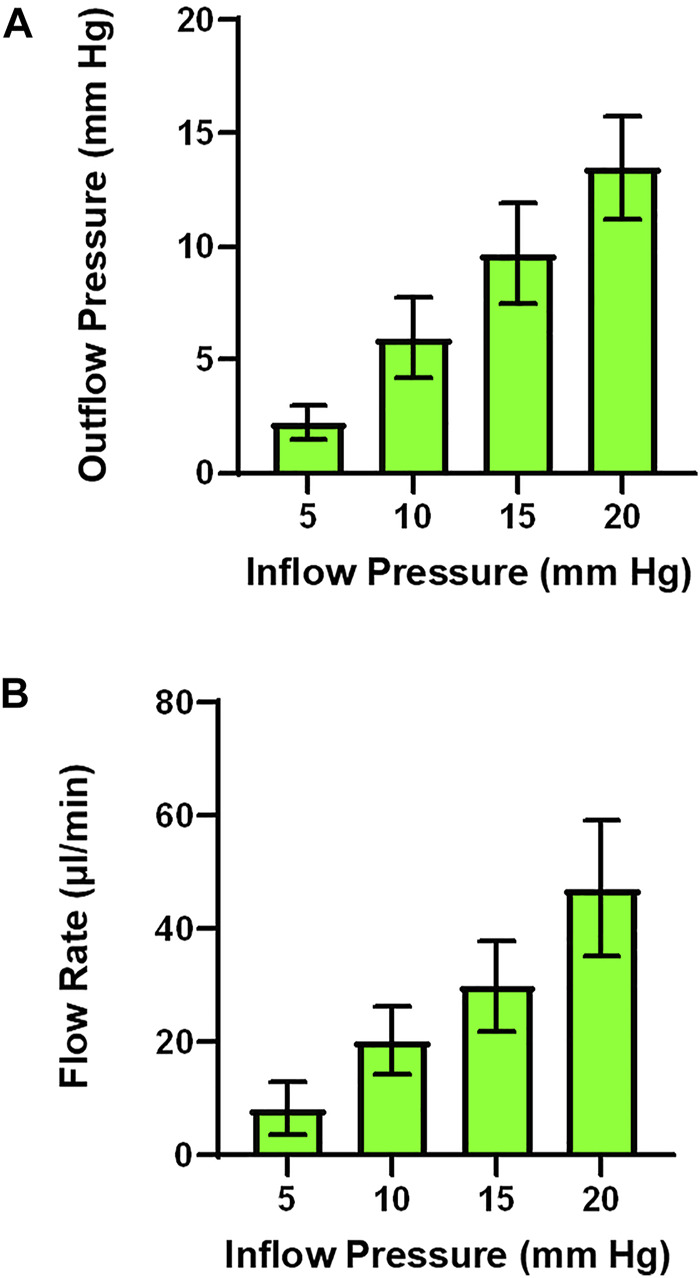
Outflow pressure and flow rate across inflow pressures. **(A)** As inflow pressure is increased in a stepwise fashion outflow pressure increases with both normal superfusate. **(B)** Alternatively, flow rate can be measured as the result of increasing inflow pressure. (*n* = 3 placentas).

Analysis Techniques and Preliminary Studies with Isolated Perfused Placenta: In Figure 5, raw data tracings are provided that establish our proficiency with this novel approach. Perfusate flow (placental input pressure) is manipulated with servo pumps in a stepwise fashion as mentioned above, umbilical artery and vein diameter can be measured simultaneously. Note that changes in pressure and flow can occur independently or in tandem. The critical functional measurements are: vascular diameters, perfusate flow, and umbilical vein pressure. Umbilical vein pressure is of greatest interest as it represents placental resistance, post-placental hemodynamic conditions, and blood flow rate returning to the fetus. The placental pressure drop (pressure delta between Figures 5A,B) is an index of vascular resistance, and therefore, placental health. The potential also exists to measure permeability and many fluorescently labeled biomarkers in the vascular wall, placental unit, effluent, and eluate. Increasing inflow pressure in the setup increases both outflow pressure (Figure 6A) and flow (Figure 6B) through the placenta. Additionally, we have previously shown that a pressure drop is observed in nano-TiO_2_ placentas (*n* = 8) compared to controls (*n* = 10) when placed in Ca^2+^-free PSS. This reflects increased resistance from an anatomical perspective (altered passive elements/structural changes) due to maternal inhalation exposure during gestation (Abukabda et al., 2019). This is important to note because increased placental resistance is inversely correlated with placental efficiency due to a decrease in vascular density and/or length within the placental circulation. Additionally, we showed decreased outflow pressure in nano-TiO_2_ placentas with normal superfusate, acetylcholine and angiotensin II treatment. Overall, we were able to conclude that maternal nano-TiO_2_ exposure during gestation impairs fetoplacental hemodynamics.

## Discussion

**FIGURE 7:**
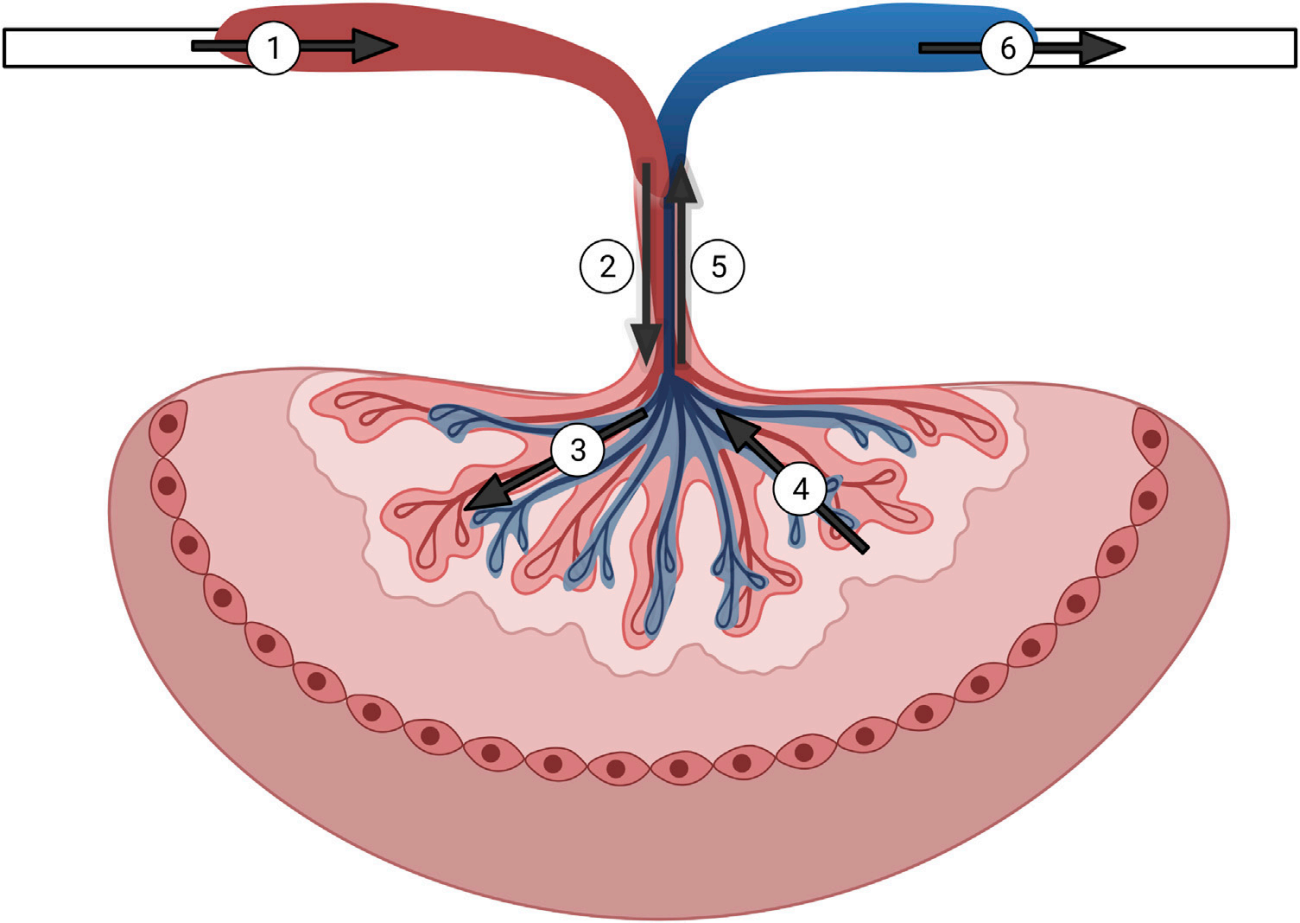
Graphical representation of the isolated rat placenta model and perfusion pathways. Numbering in the figure represents the following: 1) input pressure pipette; 2) umbilical artery; 3) arteriolar waste delivery network; 4) venular nutrient network return; 5) umbilical vein; 6) output pressure pipette. Arrows show direction of the perfusate flow.

The data presented here reflect our ability to develop a unique, reliable and efficient system to assess placental physiology within the isolated rat placenta (Figure 7). The content of this protocol was presented to demonstrate the utilitarian nature of isolated vessel chambers and repeated three times herein. We have also demonstrated the reliability of this model in previous publications (Abukabda et al., 2019). Placental health is inextricably linked to maternal and fetal health throughout gestation. Perturbations in placental development and/or blood flow throughout gestation leads to poor health outcomes for both the dam and the fetus such as preeclampsia (Brosens et al., 2002), intrauterine growth restriction (Fowden et al., 2006) and gestational diabetes (Holemans et al., 2004). Examining and understanding how disturbances in adaptations during critical windows of gestation affect fetal and maternal health is essential to improving pre- and postnatal care.

The maintenance of hemodynamic resistance is fundamental in all tissues for the healthy exchange of nutrients and wastes, and preservation of homeostasis. The fetal-placental unit is no exception. We hypothesize that toxicant exposure during gestation disturbs mechanisms of placental vascular resistance and reactivity. Specifically, impairment of endothelium-dependent reactivity would lead to an increased placental resistance, which would appear as lower post-placental pressure and flow rate. The health interpretation of this outcome would be that the fetal-placental unit is unable to efficiently remove wastes and gain nutrients leading to placental insufficiency and a smaller pup. It is equally plausible that toxicant exposure during gestation abolishes placental resistance. In that case, uterine artery and vein pressures would closely match, and again, the effective exchange of nutrients and wastes would be impaired, leading to the similar outcome of decreased pup size due to placental insufficiency. Further, the maintenance of extravascular nutrient gradients would be disturbed in the placental interstitium. Utilizing the isolated placenta as a tool to assess fetal and placental health is incredibly valuable because measuring these endpoints after specific gestational insults would advance our understanding, independent of the outcome, of the materno-fetal consequences that follow.

We anticipate two potential challenge areas. First, because the placenta is not fully developed after GD 7, the necessary umbilical vasculature for cannulation may not be present, and in our experience, it is not be possible to perfuse the placenta prior to GD 15 due to fragility and underdevelopment of the umbilical artery and vein. Second, because the placenta begins to slough cells and deteriorate rapidly after delivery, great effort must be made to prevent or slow this process. The placenta is viable for the experimental protocol for 1.5–2 h from when it is mounted in the chamber and therefore this technique must be performed rapidly. This is in comparison to the 4 hours mentioned earlier, which refers to the maximum amount of time after tissue collection that a placenta can be mounted in the chamber for experimentation. The single most important variable in doing this is to avoid a hypoxic environment that stimulates glycolysis. This can be achieved by rapid dissection, temporary placement/preparation in Ca^2+^-free PSS (4°C); and constant bubbling with 21% O_2_–5% CO_2_. In the event that this is insufficient for all the measurements proposed, the individual components can easily be separated. Additionally, these measurements could also be made in a murine model, as the model has already been established (Goeden and Bonnin, 2013). Independent of animal model, multiple placentas are present per animal, histology and fluorescence measurements, and effluent and eluate collections can be made or prepared rapidly in individual units. Lastly, caution should be taken when directly extrapolating data from this rodent model to human health given the difference in trophoblast cells at the fetal-maternal interface between the rat and human.

The isolated placenta holds the potential to examine multiple mechanisms that impair placental efficiency during gestation. Fetal size, sex, placement within the uterus, gestational age, toxicant exposure, blood flow restriction, and treatment of the placenta with circulating gestational or other factors are future areas of research that hold tremendous potential. Our data validate that this model enables the assessment of multiple endpoints within the same placenta. Additionally, by optimizing this technique we report that multiple placentas within the same dam can be utilized for experimentation.

## Data Availability

The original contributions presented in the study are included in the article/Supplementary Material, further inquiries can be directed to the corresponding author.
